# Exploring an Age Difference in Preschool Children’s Competitiveness Following a Competition

**DOI:** 10.3389/fpsyg.2018.00306

**Published:** 2018-03-09

**Authors:** Yu Hu, Yi Zhu

**Affiliations:** ^1^Research Institute of Social Development, Southwestern University of Finance and Economics, Chengdu, China; ^2^College of Psychology and Sociology, Shenzhen University, Shenzhen, China

**Keywords:** rivalry, inequality aversion, status hierarchy, willingness to compete, cognitive control

## Abstract

Literature suggests that resource acquisition compels competition in young children. However, little is still known about the development of preschool children’s competitiveness. In this preliminary study, 166 children (aged 2–4 and 5–6 years) engaged in a dyadic competition which resulted in a winning and a losing group (in a control/non-competition group, participants engaged in a similar task which did not lead to winning/losing outcome), and then experimenters tracked their decisions to compete again with a rival (i.e., an individual they interacted in the previous competition task) and a non-rival competitor (i.e., an anonymous classmate they did not interact in the previous competition task) for a reward, respectively. As expected, results showed an age-related decreasing trend in the percentage of choices to compete with a competitor. However, this age difference was only significant in the control group when participants played with the partner with whom they interacted in the previous game and in the losing group when participants competed with a non-rival competitor. This study contributes to our knowledge of how competitiveness develop in preschool childhood, and calls for further research on the roles of motivation and cognitive control in children’s competitiveness.

## Introduction

Recent years witnessed a growing body of research on people’s competitiveness. Competitiveness is considered a significant factor in explaining persistent performance gap in labor market and within organizations, especially gender difference in career development (Niederle and Vesterlund, 2007; Gneezy et al., 2009; Buser et al., 2014). Recent debates suggest that an individual difference in competitiveness may have deep roots in human life span development (Gneezy and Rustichini, 2004; Bartling et al., 2012; Cárdenas et al., 2012; Sutter and Glätzle-Rützler, 2015; Almås et al., 2016). The present research explored an age difference in preschool children’s competitiveness, with the purpose of shedding light on the development of children’s competitiveness (Charlesworth, 1996; Hawley, 1999; Green and Rechis, 2006; Pellegrini, 2008).

Children in their preschool life continuously interact with peers including their competitors, and have the chances of engaging in competitions to access to resources (Charlesworth, 1988; Pellegrini, 2008). They want to fare better than peers (Friedman, 2013; Samak, 2013; Sheskin et al., 2014; Pappert et al., 2017), and those who gain advantage in competitions usually enjoy valued resources and high group status (Strayer and Strayer, 1976; Charlesworth, 1996; Hawley, 1999; Pellegrini, 2008). Losing competitions, however, may result in negative affect (Heyman et al., 1992; Steinbeis and Singer, 2013), a poorer subsequent performance (Ruble et al., 1994; Rhodes and Brickman, 2008), and a passiveness in the face of challenges (Freniere and Charlesworth, 1987). This literature suggests that competition outcomes may have great impact on children’s behaviors. Given the ubiquity of competition in children’s preschool life, it is meaningful to study how competition outcomes would influence their competitiveness.

The topic on the development of children’s competitiveness following a competition is under explored. Several studies in economics suggest that males are more competitive than females and this gender gap has deep roots in life span development (Gneezy and Rustichini, 2004; Bartling et al., 2012; Cárdenas et al., 2012; Sutter and Glätzle-Rützler, 2015; Almås et al., 2016). Among the few research which has considered an age difference in preschool children’s competitiveness, Sutter and Glätzle-Rützler (2015) showed a gender gap in the frequency of choosing the competitive payment scheme among 5–6 year-olds, but not among 3–4 year-olds. However, their data did not show a significant age difference in competitiveness in preschoolers. Developmental psychologists view competition as a resource acqusition strategy (Charlesworth, 1988; Pellegrini, 2008). Developmental research with young children (including children at preschool age) has revealed an age-related decrease in competitiveness (e.g., insisting on priority, taking, thwarting, insulting) in resource acquisition (Strayer and Strayer, 1976; Charlesworth, 1996; Hawley, 1999; Pellegrini, 2008), however, empirical evidence on how previous competition experience affects children’s competitiveness is scant.

Therefore, we conducted a study in which 166 preschoolers (aged 2–4 and 5–6 years) engaged in a dydic competition task which resulted in a winning and losing outcomes, and after the competition task we tracked children’s decisions regarding whether they wanted to compete for an additional reward or not to competite. Hawley (1999) suggested that young children (e.g., aged 2–3 years) tend to behave competitively in resource acquisition such as insisting on priority, taking, thwarting (i.e., competitive/coercive strategies), and with the development of cognitive control and the acquisition of verbal abilities and social skills to negotiate with their peers, they also behave in prosocial manners to acquire resources such as making suggestions, helping, offering objects (i.e., prosocial strategies). Consistent with this view, recent research revealed an age-related decrease in using competitive strategies (Pellegrini et al., 2007) and increase in using prosocial strategies in resource control (Roseth et al., 2011). These literature suggests that children in early stages of the life span are incline to engage in competition for resources, and such competitiveness decreases with age. Additionally, another line of research suggests that, with age, children become more intent to consider merit and effort in resource allocation (Sigelman and Waitzman, 1991; Almås et al., 2010). In our experiment, performance in the competition task hinged on effort and ability, thus competition outcomes (i.e., winning and losing) could be more accecptable to the 5–6 year-olds, as compared with the 2–4 year-olds. Based on the above literature, we could expect an age-related decrease in the percentage of choices to compete again following a competition.

## Materials and Methods

### Participants

There were 166 native Chinese-speaking children recruited in the present study. Our sample size was determined by the number of parents provided consent and the number of children who attended classes. These participants were divided into two age groups: 79 children aged 2–4 years (*M* = 38.85 months, range = 24–55 months), and 84 children aged 5–6 years (*M* = 68.95 months, range = 60–81 months). Data from three participants in the younger age group was excluded because of their failure in following the instructions (see **Table 1**). These participants were recruited from a kindergarten in Chengdu, China. This study was approved by the ethics committee of the school.

**Table 1:** Number of participants by age, gender, and experimental conditions.

		2–4 year			5–6 year	
	
Conditions	Win	Loss	Control	Win	Loss	Control
Female	15	15	9	15	15	9
Male	12	11	17	14	14	17
All (*N* = 163)	27	26	26	29	29	26

### Competition Task

Our experimental protocol concerning the competition task was modeled from a between-group competition task used in previous studies (Zhu et al., 2015, 2016), because this task encourages fine motor skills and hand-eye coordination, and task performance (winning or losing) depends on effort and ability. An important feature of our competition task was that participants engaged in a dyadic competition. Specifically, each participant was randomly paired with another participant from the same age group. The two participants were escorted to the same experimental room by female experimenters, and one experimenter explained the rule of the task and showed the prize (a box of marshmallows) the winner would get. We used marshmallows as the prizes because these are very attractive to children as suggested by preschool teachers. The effectiveness of using candies as prizes was also proved in a previous experiment with Chinese children (Zhu et al., 2016). Each participant was led by an experimenter to a table separately in the same room (between-table distance was 3 m). On each table, there were a spoon and two containers (one contained 30 ping-pong balls and the other was empty; between-container distance was 40 cm). During the competition, the two participants transferred ping-pong balls with a spoon from one container to the other within 20 s. They were identified to be either a winner or a loser based on the number of balls they successfully transferred. The winner was rewarded a piece of marshmallow in front of the loser who received no reward. Moreover, we also included a non-competition treatment (a control group) which was identical to the task described above except that there was no dyadic competition manipulation—no information about task outcome or the prize was delivered to each child. Moreover, the participants were not allowed to observe each other’s performance in the non-competition treatment.

### Measure of Competitiveness

Similar to Samak (2013), after the competition task, participants could decide whether she/he wanted to compete again for a different prize (a piece of candy) or not to compete. Participants’ decisions were recorded by the experimenters and this binary variable (i.e., yes/no) served as our dependent variable. Because rivalry and non-rival competitive interactions are commonplace in young children (Charlesworth, 1988; Pellegrini, 2008), the experimenters instructed participants to make decision regarding whether they wanted to compete again for a prize with a rival (i.e., the same individual they interacted in the previous competition task) and a non-rival competitor (i.e., an individual participants did not interact in the previous competition task) sequentially. Note that the experimenters repeated the instructions when necessary until participants made clear decisions, and that the experiment ended without asking children to engage in a subsequent competition. All prizes were wrapped in a yellow bag and participants were instructed not to open the yellow bag and not to interact with classmates until they left the classroom.

## Results

**FIGURE 1:**
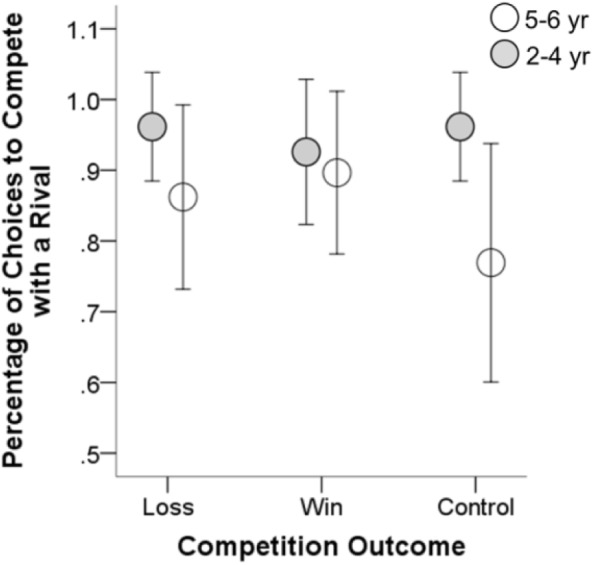
Mean percentage of choices to compete with a rival as a function of age and competition outcome. Error bars represent 95% confidence intervals.

**FIGURE 2:**
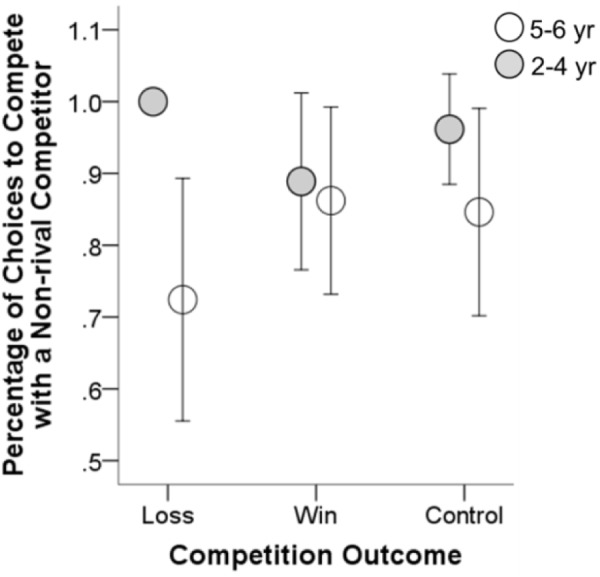
Mean percentage of choices to compete with a non-rival competitor as a function of age and competition outcome. Error bars represent 95% confidence intervals.

The mean percentage of competition choices in each group were shown in **Figures 1**, **2**. We conducted a series of statistical analyses to compare the percentage of competition choices between the 2–4 and 5–6 year-olds.

### Choice to Compete With a Rival

We first examined the age difference in children’s competitiveness when they had the choice to compete with a rival. The percentage of competition choices in the 2–4 year-olds was higher than that in the 5–6 year-olds across experimental groups, χ^2^(1, *N* = 163) = 4.725, *p* = 0.030 (see **Figure 1**). To further reveal the age difference in children’s competitiveness, we conducted a logistic regression predicting children’s competition choices (no competition = 0, competition = 1) as a function of age group (2–4 years = 0, 5–6 years = 1), controlling for gender (female = 0, male = 1), and competition outcome (loss = 0, win = 1, control = 2). Results showed a significant negative effect of age group, β = -1.252, *p* = 0.036, odd ratio [OR] = 0.286, suggesting a developmental decrease in children’s competitiveness. However, we did not observe the significant effects of gender and competition outcome.

We then conducted separate analyses in the winning, losing, and control groups. In the control group, the percentage of competition choices in the younger age group was significantly higher than that in the elder age group, χ^2^(1, *N* = 52) = 4.127, *p* = 0.042. This suggests that younger children were more intent to compete than elder children. Although age gap was also observed in the winning and losing groups, the difference was not statistically significant (*p*s ≥ 0.2), suggesting that the children of the two age groups could have similar level of competitiveness when they were to compete with a rival.

### Choice to Compete With a Non-rival Competitor

We next tested the age difference in the percentage of choices to compete with a non-rival competitor. A Chi-square test showed that there were more competition choices in the 2–4 year-olds than in the 5–6 year-olds, χ^2^(1, *N* = 163) = 7.396, *p* = 0.007 (see **Figure 2**). A logistic regression predicting children’s competition choices as a function of age group, controlling for gender and competition outcome, showed a significant negative effect of age group, β = -1.513, *p* = 0.010, OR = 0.220, suggesting that younger children are more intent to compete than their elder counterparts. Again, we did not observe significant effects of gender and competition outcome.

Separate analyses were also conducted in the control, winning and losing groups. Results showed that, in the losing group, the percentage of choices to compete with a non-rival competitor in the younger age group was significantly higher than that in the elder age group, χ^2^(1, *N* = 55) = 8.393, *p* = 0.004. The age difference was also observed in the winning and control groups (see **Figure 2**), although was not statistically significant (*p*s ≥ 0.15). Taken together, these results suggest that younger children, as compared with the elder children, were more willing to compete with a non-rival competitor, especially when they experienced a competition loss.

### Choice to Compete With a Rival vs. a Non-rival Competitor

To compare participants’ choices to compete with a rival vs. a non-rival competitor, McNemar test showed that the proportion of participants choosing to compete with a rival vs. a non-rival competitor was not significantly different, *p* = 0.710. Moreover, the difference was also not statistically significant when we conducted separate analyses in the control, winning and losing groups, *p*s > 0.500. These results suggest that competitor identity has no prominent effect on children’s competitiveness.

## Discussion

This study examined the age difference in preschool children’s competitiveness following a previous competition. Results showed an age-related decreasing trend in the percentage of choices to compete with either a rival or a non-rival competitor. Further analyses showed that this age gap was only significant in the control group when participants played with the partner with whom they interacted in the previous game and in the losing group when participants competed with a non-rival competitor.

The finding that children’s choices to compete for a reward generally decreased with age is consistent with existing literature showing that children’s use of coercive strategies (i.e., insisting on priority) in resource acquisition decreases with age (Hawley, 1999; Pellegrini et al., 2007; Pellegrini, 2008; Roseth et al., 2011). Past research on the influence of competitor’s identity (i.e., a rival or a non-rival competitor) on children’s competitiveness is scant; the present research fills this gap by suggesting that 2–4-year-old children, as compared with 5–6-year-old children, are more intent to use coercive strategies, regardless of their competitor’s identity.

It is known that cognitive control capacity is closely associated with the development of prefrontal cortex (Steinbeis and Crone, 2016). The age difference in young children’s decisions to enter competition might reflect the development of their cognitive control capacity (Hawley, 1999). As such, the 2–4-year-old children could be less capable of controlling their impulse to gain an advantage over their competitors in resource acquisition, especially in the control group and the losing group when they faced with a non-rival competitor. The non-significant age gap in the percentage of choices to compete with a rival in the winning and losing conditions might reflect the fact that encountering a rival could elicit similar level of competitive motivation to gain access to resource in the two age groups, regardless of their own performance in the competition. Similarly, the non-significant age gap in the winning group when participants faced with a non-rival competitor might suggest that winning a competition could increase younger children’s cognitive control. These speculations actually underscore the need to do further experiments to provide more insights into the roles of social context-induced motivation and cognitive control in children’s decision to compete.

Next, in one of few studies that have focused on preschoolers’ competitiveness in a dyadic competitive context, Samak (2013) showed that 80% of children chose to enter into competition. The high proportion of competition choices was also observed in the present study (see **Figures 1**, **2**). In both studies, a zero-sum rule of competition task (i.e., winners take all) could be an important factor that motivated children to enter into the competitive resource acquisition. Participants in this sort of experimental setting are deprived of the opportunity of using any prosocial strategy that has been observed in prior research (Charlesworth, 1996; Pellegrini, 2008; Roseth et al., 2011). However, in real-life situation, competition for resources ranges along a continuum from a pure *scramble competition* where everyone can access to resources to a pure *contest competition* where winners take all (Nicholson, 1954; Pellegrini, 2008). Thus, to provide a full picture of the age difference in children’s competitiveness, future study should focus on children’s competition entry in the context of scramble competition or the combination of a scramble and a contest competition.

Finally, the present study is not without any limitation. Firstly, we found that there was no significant gender difference in terms of decisions to compete. Because we did not consider the potential effect of competitor’s gender on children’s decision, it is still unclear whether gender difference could be observed in the mixed-gender dyadic competition or in the same-gender dyadic competition. Secondly, it is likely that fewer 5–6-year-old children wanted to compete because they were not interested in this game anymore, or because 2–4-year-old children just wanted to earn a prize. Thus future study should add a control question asking children whether they wanted to play a game that does not involve competition. Thirdly, during the experiment, our experimenters recorded participants’ decisions regarding whether they wanted to enter a subsequent competition. The presence of our experimenters could have a subtle effect of affecting children’s decisions. Additionally, this research did not document information such as children’s family background (Almås et al., 2016) and attachment styles (Chen and Chang, 2012; Chen, 2015) which may have profound impact on children’s competitiveness. Finally, this study examined preschool children’s competitiveness, in order to better picture the development of human competitive behavior, future research should include more age groups (e.g., 7–8 and 11–12 years). Therefore, we believe that it is important to take at least these five issues into account in the future research.

In summary, our study provides preliminary results about the age difference in preschool children’s competitiveness. Results showed a developmental decrease in the percentage of choices to compete, especially in the control group and the losing group where participants faced with a non-rival competitor. Our findings may stimulate more empirical and theoretical work on this topic in future studies.

## Ethics Statement

This study was carried out in accordance with the recommendations of Ethics Committee of Shenzhen University with written informed consent from children’s parents. All children’s parents gave written informed consent in accordance with the Declaration of Helsinki. The protocol was approved by the Ethics Committee of Shenzhen University.

## Author Contributions

YH and YZ conceived of the study and collected the data. YZ analyzed the data and wrote the paper.

## Conflict of Interest Statement

The authors declare that the research was conducted in the absence of any commercial or financial relationships that could be construed as a potential conflict of interest.
